# Editorial: 14-3-3 proteins: possible importance in neurodegenerative diseases

**DOI:** 10.3389/fmolb.2024.1525126

**Published:** 2024-12-06

**Authors:** Dwijendra K. Gupta, Alan M. Tartakoff, Manish Dwivedi

**Affiliations:** ^1^ Department of Biochemistry, Central University of Allahabad, Allahabad, India; ^2^ Departments of Pathology, Genetics and Genome Sciences, Biochemistry and Molecular Biology & Microbiology, School of Medicine, Case Western Reserve University, Cleveland, OH, United States; ^3^ Amity Institute of Biotechnology, Amity University Uttar Pradesh, Lucknow, India; ^4^ Research Cell, Amity University Uttar Pradesh, Lucknow, India

**Keywords:** neurodegenerative diseases, 14-3-3 proteins, dementia, therapeutics, alzeimer’s disease

The conversion of proteins from their native state into well-organized fibrillar aggregates, whether inside cells or in the extracellular space of connective tissue, leads to the pathogenesis of several human diseases that include neurodegenerative diseases as well as non-neuropathic conditions, such as systemic amyloidosis. Neurodegenerative diseases including Alzheimer’s disease (AD) Parkinson’s disease, and Huntington’s disease are marked by personality changes, memory loss (dementia), cognitive changes, blurred vision, insomnia, and loss of motor control. Despite substantial advances, these disorders are usually fatal or lead- to serious debilitation and dramatic worsening of the quality of life. As a result, there is a widely appreciated need to further elucidate the pathogenesis of these diseases and to develop effective therapies. Significant progress in elucidating molecular mechanisms that trigger the aggregation and the pathogenicity of proteins has enabled the design of novel and potentially powerful treatments. Nevertheless, little is known of the critical interactions in the complex *in-vivo milieu* which determines where and when protein deposition occurs in individual patients. Moreover, understanding of which molecular species are responsible for pathogenesis is far from complete.

14-3-3 proteins are small, conserved scaffolding molecules that are expressed in all eukaryotes. They modulate the function of other proteins, primarily in a phosphorylation-dependent manner. They are abundantly expressed in the brain and are often thought to be involved in age-related neurodegenerative diseases. To date, several hundreds of 14-3-3 binding partners have been identified. This diverse group includes protein kinases, phosphatases, receptors, transcription factors, structural and cytoskeletal proteins, as well as small G-proteins and their regulators. Consequently, 14-3-3 proteins could be promising targets for therapeutic intervention. Previous studies on invertebrates have demonstrated the importance of these proteins for regulation of synaptic function, learning and memory. Nevertheless, the exact roles of 14-3-3 proteins in neurodegenerative diseases are still unclear. The present Research Topic of articles brings together original research, reviews, and perspectives in the biology of 14-3-3 proteins and their possible relevance for therapy of neurodegenerative disease.

An article by Obsilova and Obsil describes the molecular mechanisms through which 14-3-3 proteins regulate their binding partners. These mechanisms fall into three categories: 1) direct conformational modulation of the bound partner; 2) physical occlusion of sequence-specific or structural features on the surface of the target protein; and 3) scaffolding, which facilitates protein-protein interactions (Obsilova and Obsil).

Although most binding partners of 14-3-3 proteins contain phosphorylated binding motifs, these proteins can also bind to non-phosphorylated motifs with high affinity. One example of phosphorylation-independent interaction is with exoenzyme S (ExoS), a bacterial ADP-ribosyltransferase toxin of *Pseudomonas aeruginosa*. This interaction is essential for toxin function. Structural studies on the complex between 14-3-3ζ and the ExoS peptide, identified as an amphipathic motif, revealed that the ExoS peptide binds to 14-3-3 mainly through hydrophobic contacts (Ottmann et al., 2007a). Another well-characterized peptide, the R18 peptide, derived from a phage display library, binds 14-3-3 proteins with high affinity through an amphipathic motif (Wang et al., 1999).

14-3-3 proteins modulate the activity of many enzymes including arylalkylamine N-acetyltransferase, tyrosine hydroxylase, tryptophan hydroxylase, yeast neutral trehalase, apoptosis signal-regulating kinases, protein kinases B-RAF and C-RAF, leucine-rich repeat protein kinase-2 (LRRK2), protein kinase C (PKC), calcium/calmodulin-dependent protein kinase kinases, death-associated protein kinase 2, phosphatidylinositol-4-kinase-III, protein phosphatase CDC25C, and E3 ligase neural precursor cell expressed developmentally downregulated 4 ligase.

Interestingly, 14-3-3 protein binding motifs, are often located near a nuclear localization sequence (NLS) or nuclear export sequence (NES) and can thereby modulate the subcellular localization of its target (Muslin and Xing, 2000). A well-known example is Forkhead box O transcription factors (FOXO), which are crucial for cell survival, DNA damage repair, and stress resistance (Gui and Burgering, 2022). Other examples are Class II histone deacetylases (HDAC4 and HDAC5), whose subcellular localization is controlled in a phosphorylation-dependent and 14-3-3 dependent manner through two 14-3-3 binding motifs: a NLS located between these two motifs, and a NES at the C-terminus, which is inactive in unphosphorylated HDACs.

Obsilova and Obsil have shown that 14-3-3 proteins contribute to CaMKKs (both CaMKK1 and CaMKK2) inhibition by protecting the inhibitory phosphorylation sites from dephosphorylation. A similar mechanism also contributes to 14-3-3-dependent inhibition of CaM-regulated Ser/Thr protein kinase DAPK2, which is involved in apoptosis, autophagy, granulocyte differentiation and motility regulation (Bialik and Kimchi, 2006). DAPK2 kinase activity is suppressed through autoinhibition, homodimerization and 14-3-3 binding to its C-terminal canonical mode III phosphorylated motif (Gilad et al., 2014; Yuasa et al., 2015). In their biophysical studies on the interaction between autophosphorylated DAPK2 and 14-3-3γ, Obsilova and Obsil have observed that the formation of the complex stabilizes DAPK2 dimerization, protects against DAPK2 inhibitory autophosphorylation and suppresses Ca^2+^/CaM binding (Horvath et al., 2021).

In addition to phosphorylation-specific interactions, 14-3-3 proteins exhibit phosphorylation- and ATP-independent chaperone-like activity [reviewed in Sluchanko and Gusev (2017)] that apparently prevents aggregation of partly folded or misfolded proteins This activity is greater in monomeric forms of 14-3-3 proteins, possibly due to the exposure of hydrophobic residues (Sluchanko et al., 2011). Furthermore, 14-3-3 proteins regulate small heat shock proteins (HSPB6) by stabilizing its intrinsically disordered N-terminal domain (Sluchanko et al., 2017). This interaction seems to be essential for smoot muscle contraction (Dreiza et al., 2005). The chaperone activity of 14-3-3 proteins is also involved in the regulation of *Pseudomonas* exotoxin-S and -T (ExoS and ExoT), by protecting against their thermal aggregation. Thus, 14-3-3 proteins may activate ExoS and ExoT by protecting their hydrophobic surfaces from aggregation with α-synuclein (Plotegher et al., 2014). Conversely, 14-3-3 proteins stimulate the aggregation of unphosphorylated Tau protein, a neuronal protein involved in microtubule stabilization and a major component of intraneuronal neurofibrillary tangles in patients with AD (Sadik et al., 2009; Sluchanko and Gusev, 2011; Qureshi et al., 2013; Neves et al., 2021).

Other roles of 14-3-3 proteins are stabilization of the oligomeric state of target proteins and anchoring of different proteins near to each other. A paradigmatic example of such a mechanism is the activation of plant plasma membrane H+ -ATPase (Ottmann et al., 2007b). Modulation of 14-3-3 proteins therefore may be is a promising strategy for treating these pathologies.

A thorough proteomic analysis using multistep immunoaffinity purification and mass spectrometry has identified 271 yeast proteins that specifically interact with their 14-3-3 proteins (Bmh1, Bmh2) in a phosphorylation-dependent manner (Kakiuchi et al., 2007). Yeasts, unlike higher eukaryotes, usually express only one or two 14-3-3 protein isoforms. In a further article, Obsilova and Obsil describe how cell signaling regulates physiological processes by receiving, processing, and transmitting signals between the extracellular and intracellular environments. The targets here include catabolite repression, carbon metabolism, endocytosis, and mitochondrial retrograde signaling.

The article by Awasthi et al. concerns the possible dependence of AD on viral infection of B lymphocytes, e.g., with Epstein-Barr virus. This group has emphasized co-expression analysis of genes and has identified seven genes (YWHAH, YWHAG, YWHAB, YWHAZ, MAO2K1, PP2CA and TUBB) that show similar expression patterns in various human brain regions. Apoptosis and aspects of cytoskeletal organization correlate with the expression of these genes. Furthermore, three potentially regulatory miRNAs (hsa-mir-15a-5p, hsa-let-7a-5p, and hsa-mir-7-5p) were identified. These miRNAs may serve as biomarkers for AD linked to viral infections. On the basis of these findings, the authors also identify drugs that could play a significant role for treatment of AD (Awasthi et al.). The article of Abdi et al. further examines the function of multiple 14-3-3 proteins in neurological disorders, with emphasis on AD (Abdi et al.).
